# Histological Investigation of the Cleaning Effectiveness of Different Biomechanic Processes of Isthmus in Lower Molars

**DOI:** 10.1055/s-0042-1753455

**Published:** 2022-11-09

**Authors:** Rabia N. Aydın, Nimet Gençoğlu

**Affiliations:** 1 Department of Endodontics, Faculty of Dentistry, Istanbul Medipol University, Istanbul, Turkey; 2 Department of Endodontics, Faculty of Dentistry, Marmara University, Istanbul, Turkey

**Keywords:** debris, isthmus, sonic irrigation, ultrasonic irrigation

## Abstract

**Objectives** This study aimed to evaluate the efficacy of different instruments (Hyflex, ROTATE, and hand files) and irrigation methods (EndoUltra, EndoActivator, and side-perforated syringe) in the mesial root canal of a mandibular first molar with isthmuses.

**Materials and Methods** Sixty-three mandibular molar teeth with isthmus were selected using cone beam computed tomography (CBCT) images. The root canals were instrumented with Hyflex, ROTATE, or hand files (21 of each) and three of each as control group and the specimens were embedded in silicone blocks. Final irrigation was performed with the perforated syringe or ultrasonic (EndoUltra) or sonic irrigation (EndoActivator). All canals were irrigated using 5.25% NaOCl solution (15 mL). Then, the roots were stained with hematoxylin–eosin, and specimens were sliced for histologic evaluation. The isthmus regions (2.7, 3, 4.7, and 5 mm from the apex) were analyzed, and the percentage of debris was calculated.

**Statistical Analysis** All data were statistically analyzed using one-way analysis of variance and Tukey's tests.

**Results** Both Hyflex and ROTATE groups showed less debris compared with the hand instrument group ( *P* < 0.05). When irrigation methods were compared, EndoUltra showed the least, followed by EndoActivator and perforated needle irrigation having the maximum debris ( *p* < 0.05). When sections were compared, the maximum debris was found in the apical section and the least in the coronal section ( *p* < 0.05).

**Conclusion** Although none of the methods cleaned the isthmus completely, nickel–titanium (NiTi) systems and ultrasonic and sonic systems removed more debris than the side-perforated needle syringe irrigation.

## Introduction

The outcome of endodontic treatment depends on the elimination of vital and necrotic tissues, microorganisms, and their products. However, this treatment is difficult to achieve in the anatomical complexes, such as isthmus, lateral canals, and anastomosis, that are frequently inaccessible to the instrument. 1

Studies have shown that 9.6 to 48% of the main root canals remain unshaped after instrumentation. 2 3 4 In the anatomical complex, the isthmus is defined as a narrow communication between two canals which contains microorganisms and dentin debris resulting from instrumentation. 5 It is difficult to access the isthmus area; it may lead to the failure of endodontic treatment when proper instrumentation and cleaning are not achieved.

The mandibular first molars are the most treated teeth endodontically due to the early age of eruption. Further, 55% of mesial roots and 20% of distal roots of mandibular molars contain isthmus communications, usually in areas 3 to 5 mm away from the apex. 6 The untouched areas are present after instrumentation. Hence, irrigation plays an important role in cleaning and disinfecting the isthmus. 7 8 Many methods have been used to enhance the delivery of the irrigation solution to mechanically inaccessible areas of root canal systems, ranging from conventional syringe irrigation to sonic or ultrasonic systems. The needle irrigation technique is widely used; however, the distribution of solutions within the apical areas, such as isthmuses, is difficult. Also, the penetration of the irrigant depends on the distance of the needle tip to the working length, flow rate, and needle design. 9 Previous studies showed that the side-vented needle irrigation resulted in better performance compared with conventional needle irrigation. 10 Also, ultrasonic or sonic activation systems have been proposed to activate the irrigant for better cleaning and disinfection of the root canal system.

Sonic irrigation devices work at a low frequency (1–6 kHz) than ultrasonics (25–30 kHz) and produce smaller shear stresses. 11 The sonic energy generates significantly higher amplitude or greater back-and-forth tip movement. 8 EndoActivator (Dentsply Maillefer, Ballaigues, Switzerland) is one of the sonic devices with flexible polymer tips which can be used for agitating the irrigation solution within the root canal to clean canal ramifications. It is found to be effective in removing debris or smear layer. 12 13 14

Ultrasonic systems can also be used for agitating irrigation solutions. The cleaning action is attributed to cavitation and acoustic streaming. 8 Most studies indicated that ultrasonic activation was more effective than conventional irrigation methods or sonic systems in terms of debris removal. 13 15

EndoUltra is the only cordless ultrasonic handpiece with a unique multiuse activator tip working at a frequency of 40,000 Hz. No study reported on EndoUltra performance regarding debris removal in the isthmuses.

Nickel–titanium (NiTi) devices mostly work with the crown-down technique and remove debris coronally, thus reducing the accumulation of debris more than hand instruments. Also, it is claimed that greater taper instruments remove more debris than hand instruments. 16 17

Hyflex EDM files are produced from a controlled-memory alloy using regenerative technology. It has been stated that the mechanical properties are considerably improved with superior breaking resistance and cutting efficiency. 18

Another rotating file system, ROTATE (VDW GmbH, Munich, Germany), has an **S** -shaped section and consists of three basic files (15/0.04; 20/0.05; and 25/0.04). It has higher cutting efficiency due to its cross-sectional shape, and hence provides effective removal of debris and is suitable for the preparation of narrow and curved canals. 19

This study aimed to evaluate the efficacy of different instrumentation techniques and irrigation methods in mesial canals with isthmus in mandibular molars histologically.

The null hypothesis was that no difference existed between instrumentation and irrigation techniques in terms of the ability to remove debris from the isthmus of mandibular mesial canals.

## Materials and Methods

### Specimen Selection

This study was performed following a protocol approved by the ethics committee of our faculty (27.06.2019, 2019–335). Recently extracted noncarious mandibular molars for periodontal reasons were used ( *n* = 100). The teeth were randomly placed using a computer-generated randomization in close contact with each other as individual samples in a silicone-based impression material to simulate their natural alignment in the dental arch. Models were scanned with a cone beam computed tomography (CBCT) device (SIDEXIS 4 software; Dentsply Sirona, Pennsylvania, United States) to distinguish roots with a continuous isthmus in the middle and apical third of the roots (90 kV, 10 Ma, 0.20-mm slice thickness). Further, 70 teeth met the criteria and were used; seven teeth served as the control group for histologic evaluation.

### Root Canal Preparation

The mesial canals of all specimens were instrumented by one endodontist experienced with all methods tested. The three experimental groups represented the three irrigation techniques applied after the last instrument used. After access cavity preparation, a no. 10 K file (Dentsply, Switzerland) was inserted into the root canal and the working length was established 0.5-mm short of the foramen. During instrumentation, 2 mL of 5.25% NaOCl solution was used after the application of each file, and the same volume of solution was used for final irrigation in all groups.

Group 1 ( *n* = 21): the root canals were instrumented with Hyflex EDM 25/∼ (HEDM; Coltene/Whaledent, Altstätten, Switzerland).
Group 2 ( *n* = 21): the root canals were instrumented with a ROTATE system (VDW GmbH, Munich, Germany) using 15.04, 20.05, and 25.04 files.
Group 3 ( *n* = 21): the root canals were instrumented with nos. 15, 20, 25, and 30 K-files.

Specimens from each group were randomly assigned to one of the following three groups ( *n* = 7) for final irrigation protocols with 2 mL of 5.25% NaOCl following the manufacturer's protocols.

EndoUltra: each irrigant was activated using EndoUltra (Micro-Mega, Besançon Cedex, France) for 60 seconds at 40 kHz, using a no. 15/02 metal activator tip in an up-and-down motion.
EndoActivator: each irrigant in the second group was activated using the EndoActivator (Dentsply, Maillefer, Switzerland) for 60 seconds at 10,000 cycles per minute (167 Hz) using a no. 25/04 polymer tip inserted 1-mm short of the working length in a circular axial movement.

The last group was irrigated by placing a 30-G side-vented needle (Navitip, Ultradent Products Inc., Utah, United States) 2-mm away from the apical foramen passively into the canal.

### Histologic Evaluation

After completion of irrigation protocols, the samples were fixed with 10% neutral buffered formalin for 48 hours and then demineralized for 7 to 10 days using commercial decalcification solution (Osteomoll Merck, Massachusetts, United States). Subsequently, the samples were rinsed with water and dehydrated by passing through a series of alcohol (70, 90, 95, and 100%), cleared with xylene, and embedded in paraffin for histological evaluation. Serial sections of 5-µm thickness were obtained from each root segment with a rotary microtome, transferred to a slide, and stained with hematoxylin and eosin. Each of the sections was selected at four levels (i.e., 2.7, 3, 4.7, and 5 mm from the apex), and the stained sections were examined using a photomicroscope (Olympus Bx51, Tokyo, Japan) with ×4 objective and photographed with a charge-coupled device (CCD) camera (Olympus DP 72, Tokyo, Japan). Image analysis and processing were performed using image analysis software (ImageJ; National Institutes of Health, Maryland, United States). The outlines of the mesiobuccal and mesiolingual canals and the isthmus were traced to determine the surface area of the respective regions. The areas occupied by debris in the relevant regions were also determined. For each canal and isthmus level, the percent area occupied by the debris area was calculated by dividing the area of debris by the sum of the corresponding canals or isthmus area. Evaluations were performed by an operator blinded to the experimental groups.

### Statistical Analyses

The sample size was determined on the basis of an effect size ( *f* ) of 2.01, an *α* of 0.05, a power of 0.95, and the number of groups. Power analysis suggested that seven samples would be adequate for effective measurement of debris. All data obtained from this study were statistically evaluated using the IBM SPSS Statistics 22 (IBM SPSS, Turkey) program. The suitability of the parameters to normal distribution was evaluated with Kolmogorov–Smirnov and Shapiro–Wilks tests, and they were found to be suitable for normal distribution. Three-way analysis of variance (ANOVA) was used to evaluate the effect of sectioning, preparation, and irrigation methods on the percentage of debris. In post hoc analyses, Tukey's honestly significant difference (HSD) test was used when the variances of the groups were homogeneous, and Tamhane's T2 test was used when they were not homogeneous. In addition, one-way ANOVA tests (post hoc Tukey's HSD and Tamhane's T2 test) were used as the continuation tests ( *p* < 0.05).

## Results

**Fig. 1:**
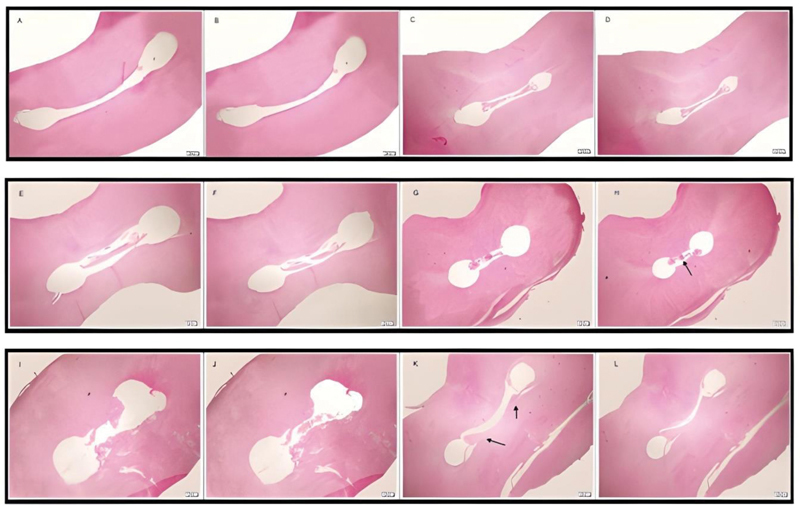
Representative photomicrographs of group 1 are shown. ( **A** – **D** ) EndoUltra group: ( **A** ) 5, ( **B** ) 4.7, ( **C** ) 3, and ( **D** ) 2.7 mm ( **E** – **H** ) EndoActivator group ( **E** ) 5, ( **F** ) 4.7, ( **G** ) 3, and ( **H** ) 2.7 mm. ( **I–L** ) Side-perforated needle group: ( **I** ) 5, ( **J** ) 4.7, ( **K** ) 3, and ( **L** ) 2.7 mm. Hematoxylin and eosin stain; magnification scale: 200 µm. The arrow indicates the debris accumulation.

Fig. 1A–L shows photomicrographs illustrating canal and isthmus cleanliness achieved by EndoUltra, EndoActivator, and side-vented needle after Hyflex preparation at different root levels.

**Fig. 2:**
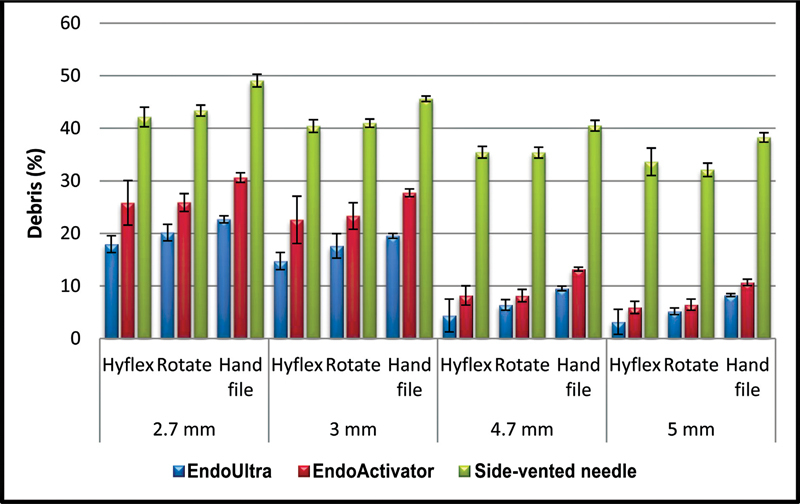
The distribution of the amount of debris according to the irrigation method of the groups.

**Fig. 3:**
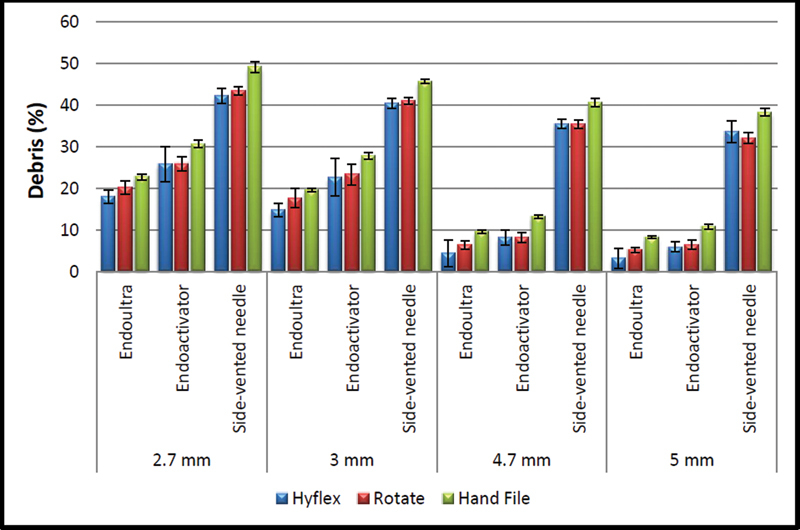
The distribution of the amount of debris according to the instrumentation method of the groups.

Figs. 2 and 3 show the percent area occupied by debris in the isthmus in each experimental group.

The data analysis showed that significantly more debris was detected in the apical sections (2.7 and 3 mm) than coronal sections (4.7 and 5 mm), regardless of irrigation and preparation techniques. No other difference was found between groups related to levels ( *p* > 0.05).

When instrumentation techniques were compared, both Hyflex and ROTATE groups showed statistically less debris than the hand instrument group ( *p* < 0.05) at all root levels. However, no significant difference was found between the ROTATE and Hyflex groups ( *p* > 0.05).

Also, a statistically significant difference was found among irrigation methods at all root levels in terms of isthmus cleanliness. The side-perforated needle group showed statistically more debris than EndoUltra or EndoActivator groups ( *p* < 0.05), and the EndoUltra group showed the least amount of debris ( *p* < 0.05).

## Discussion

In the present study, clinically most used instrumentation (NiTi files or hand files) and irrigation techniques (ultrasonic, sonic, or side-vented needle) were compared in terms of the cleanliness of debris in isthmuses during the instrumentation of mandibular mesial canals histologically. The debris may contain infected pulp or dentin tissue remnants, and hence the accumulation of debris in canal fins and isthmuses can be clinically important for the success of the root canal treatment.

Previously published studies showed that the root canal treatment in molar teeth resulted in lower success than in anterior teeth. 20 One of the reasons for the failure could be related to untouched or uncleaned areas such as isthmus or lateral canals. Therefore, different instruments and irrigation techniques were used to assess which technique or instrument would perform better cleanliness.

Paqué et al investigated debris accumulation in the isthmus after instrumentation without irrigation and found that 39 to 43% of the canal system and apical portals of exits were filled with debris which was significantly more than that before instrumentation. 1 This result indicated that the canal instrument removed and transported debris into the canal recession. Yang et al also found 39 to 42% untreated areas after preparation in mesial canals of mandibular molars. 21 These findings might be associated with the kinematics of the instrument (pecking motion) and also irregularities of the canal shape. However, another study investigated the irrigation effect on the isthmus and found that the use of 1% NaOCl irrigation left approximately 7% accumulated debris, but this amount decreased to 3.7% after passive irrigation use. 22 It was claimed that half of the debris that accumulated during instrumentation remained in the root canal. Most study results showed that NiTi instruments shaped better and removed more debris than hand instruments owing to their taper and design. 16 17 In the present study, both NiTi instruments removed more debris than the hand instrument, and therefore the null hypothesis was rejected. Meanwhile, none of the instruments completely removed debris in isthmuses. These findings were consistent with previously published study results. 23 24 25 Albrecht et al claimed that a wider taper design of the instrument might remove more dentin. 25 Da Frota et al obtained better results with ProTaper than with hand files in terms of debris removal in their histological study. 23 Devi et al achieved less debris with Hyflex EDM than ProTaper and hand instruments. 26 Safa also declared better results with Hyflex EDM compared with 2Shape systems. 27 Therefore, two NiTi instruments with different taper designs were compared in the present study. Although Hyflex instrument had a square shape in the apical section, trapezoid shape in the middle section, and triangular shape in the coronal section with a wider taper (8% in apical 4 mm.) and removed more debris than the ROTATE (4%) instrument, the difference was not statistically significant.

It is mostly difficult to reach canal intricacies such as isthmus or anastomosis by instrumentation. Hence, irrigation plays an important role in cleaning and disinfecting unreached canal sections. Most studies showed that the activation of irrigation improved the cleanliness of the canal and isthmus areas. Therefore, sonic and ultrasonic irrigations were used and compared with side-vented irrigation in the present study.

Adcock et al and other researchers declared that the activation techniques were effective in the cleanliness of the root canal in molars, but data on the complete cleanliness of the isthmus were lacking. 28 Therefore, only the isthmus area was examined in the present study. CBCT isthmus prevalence was found to be the least in the apical 1-mm zone (17.24%) and the maximum (85%) in the apical 5-mm zone. Hence, sections 2.7 to 3 and 5 mm away from the apical area were evaluated with regard to CBCT images of the selected teeth.

CBCT was used in our study to ensure isthmus standardization because it was possible to visualize the isthmus longitudinally from the pulpal ceiling to the apical area with CBCT. 29 The use of CBCT not only represented a noninvasive methodology but also allowed the longitudinal location of images, increasing their precision. Periapical radiographs provide limited opportunity to visualize the isthmus area, while CBCT images can show the isthmus with high sensitivity. In addition, a strong correlation has been found between the reconstructions of CBCT and the histological sections of the same teeth. 30

Conventional needle irrigation is the most used technique clinically. However, Munoz and Camacho-Cuadra declared that the conventional needle failed to deliver irrigation solutions into the intricate areas of the canal. 31 Goldman et al found that the side-vented needle irrigation demonstrated better performance than conventional needle irrigation. 10 Therefore, in the present study, the side-vented needle was used, but both sonic (EndoActivator) and ultrasonic (EndoUltra) systems were found to be superior to the side-vented needle irrigation. Also, the results of most studies were consistent with our findings. 29 31 32 Also, van der Sluis et al indicated that passive ultrasonic systems were more effective in removing organic tissue planktonic bacteria or infected dentin than conventional needle irrigation. 15 When sonic and ultrasonic systems were compared, conflicting results were obtained. It was claimed that passive ultrasonic irrigation (PUI) produced cleaner canals than passive sonic irrigation due to acoustic streaming and cavitation produced by the ultrasonically activated file. 33 Sabins et al and Çapar and Aydinbelge reported that PUI produced significantly cleaner canals than passive sonic irrigation. 34 35 However, Rodig et al demonstrated greater smear layer removal using the EndoActivator than ultrasonic agitation and a canal brush. 15

Studies have shown that mechanical flushing plays an important role in cleaning the isthmus. Different mechanical systems with different flow rates were used in previous studies, affecting the results. Sonic systems produce 1- to 6-kHz vibration, while ultrasonic systems produce 25 to 30 kHz. In the present study, EndoUltra ultrasonic handpiece was used which had a fine NiTi tip oscillating at a frequency of 40 kHz which moved without touching the dentinal wall in the apical area.

Ballal et al compared smear layer removal in the incisor teeth and found that EndoUltra was more effective in the apical area than the side-vented or EndoSafe irrigation system. 36 Karade et al achieved significantly better cleanliness with EndoUltra than with EndoActivator in the apical third areas of the premolar. 37 Sartiono and Iskandar also found EndoUltra effective in debris removal in the apical third area of mandibular premolars. 38 However, no other study was reported regarding the cleanliness effect of EndoUltra in the isthmus.

Sonic EndoActivator has a plastic tip and works with an apical negative pressure approach. In the present study, statistically less debris was detected with EndoUltra compared with EndoActivator ( *p* < 0.05). Both sonic and endosonic systems seemed to have a better cleanliness effect than hand irrigation. Also, EndoUltra, EndoActivator, side-vented needles were found to be 88, 83, and 60% effective, respectively, regarding debris removal in the isthmus in mesial roots of mandibular molars. Different results were obtained in previous studies depending on methodologic variations, such as application time, location of needle insertion, solution volume and concentrations, evaluation methods, teeth samples, or scoring system.

When the location of debris was evaluated, almost all study results showed that the apical section contained more debris than the middle or coronal section. 39 40 These findings were consistent with our findings showing statistically more debris in apical sections in all groups at distances of 2.7 and 3 to 4.7 to 5 mm. These results indicated that the instruments not only were ineffective in removing whole debris in the isthmus but might also transport debris back to the apical area.

In the present study, a closed-canal system was used to simulate the clinical situation and evaluate the effect of vapor lock on the root canal debridement.

## Conclusion

In the present study, NiTi instruments (Hyflex or ROTATE) were found to be superior to hand instruments regarding the debris removal from the isthmus. The ultrasonic (EndoUltra) handpiece was superior to the sonic (EndoActivator) system, and both systems showed better performance than the side-vented irrigation in all sections. However, none of them could remove whole debris in apical sections at a distance of 2.7 mm.
